# Exploring Biomarkers in Congenital Heart Disease: A Case–Control Study of ST2 in Children with Atrial Septal Defects

**DOI:** 10.3390/ijms27083445

**Published:** 2026-04-12

**Authors:** Henning Clausen, Elin Friberg, Mikko Sairanen, Pia Sjöberg, Petru Liuba

**Affiliations:** 1 Pediatric Cardiology, Children’s Heart Centre, Skåne University Hospital, 221 85 Lund, Sweden; 2 Pediatrics, Department of Clinical Sciences Lund, Lund University, 221 00 Lund, Sweden; 3 Research and Development Division, Revvity, 20520 Turku, Finland; 4 Department of Clinical Physiology, Skåne University Hospital, 221 85 Lund, Sweden; 5 Clinical Physiology, Department of Clinical Sciences, Lund University, 221 84 Lund, Sweden

**Keywords:** ST2, soluble growth stimulation protein form of interleukin-1 receptor-like 1, atrial septal defect, cardiac magnetic resonance, children, congenital heart disease

## Abstract

Soluble growth stimulation protein form of interleukin-1 receptor-like 1 (ST2) may signal myocardial stress, and elevated ST2 blood levels are associated with adverse outcomes in adult heart disease. Data on ST2 in children with congenital heart disease (CHD) is limited. This study explored ST2 in newborns and older children with atrial septal defect (ASD), as this represents a common CHD type that remains clinically challenging to recognize in childhood with slowly evolving symptoms. A case–control study was carried out in newborn ASD cases versus controls measuring ST2 on dried blood spot samples and additionally in pediatric ASD cases versus controls on venous blood together with cardiac magnetic resonance before and after treatment. ST2 was higher in newborns with ASD (*n* = 19) compared to controls (*n* = 93); (*p* < 0.01). Receiver operating characteristics to diagnose newborn ASD by ST2 showed an area under the curve of 0.848. Levels of ST2 decreased in pediatric ASD (*n* = 16) after treatment (*p* = 0.014). Lower left ventricular ejection fraction correlated with higher ST2 levels before (*r* = −0.348) and after treatment (*r* = −0.497). Elevated ST2 in newborns may aid early ASD diagnosis. Levels of ST2 in pediatric ASD decrease after treatment, and higher levels are associated with lower left ventricular ejection fraction, warranting further study.

## 1. Introduction

**Noteworthy Findings**

- In newborns with ASD, dried blood spot levels of ST2 are higher than in controls, which could aid early diagnosis.
- In children with ASD, venous blood levels of ST2 decrease following treatment, and higher ST2 levels correlate with lower left ventricular ejection fraction, which may reflect changes to cardiac loading conditions and may allow for identification of cases at risk of evolving left ventricular dysfunction.

Soluble growth stimulation protein form of interleukin-1 receptor-like 1 (ST2) is part of the interleukin-1 cytokine family and helps to balance inflammatory processes by functioning as a decoy receptor for the ‘alarmin’ interleukin-33 (IL-33) throughout the body [1]. Increased ST2 levels are noted under cardiac stress, which may lead to cardiac fibroblast activation, cellular promotion of collagen synthesis and ultimately cardiac fibrosis with associated myocardial dysfunction [2,3,4,5]. Production of ST2 may also originate from the pulmonary vascular endothelium and type 2 pneumocytes, highlighting the interdependence between the heart and pulmonary circulation [6,7]. The effects of the ST2/IL-33 axis on the cardiovascular system are due to upregulated soluble ST2, which functions as a decoy receptor for IL-33, leading to reduced IL-33 levels; this limits the cardioprotective effects of IL-33 and increases the risk for cardiac fibrosis and remodeling of the heart with an increased risk of developing heart failure [8,9,10,11]. When the heart is exposed to biomechanical stress, soluble ST2 may be secreted by the lungs and cardiomyocytes [7,10]. In ASD patients, such stress on the heart may be due to increases in right-sided volumes, with increased pulmonary artery blood flows and increased ST2 levels being noted in untreated adult ASD patients with increased pulmonary arterial pressures [12]. To date, no previous studies have investigated ST2 in young children with ASD and compared results before and after treatment to cardiac magnetic resonance (CMR) imaging findings. In this context, ST2 levels may offer additional value compared to other commonly studied cardiovascular biomarkers, such as natriuretic peptides including amino-terminal prohormone of brain natriuretic peptide (NT-proBNP). The cardiovascular biomarker NT-proBNP may correlate with right-sided heart pressures, pulmonary artery pressures and atrial shunt ratios in adult ASD [13]; however, levels may be within normal range before ASD treatment, which may limit the usefulness of NT-proBNP in this setting [14]. In previous pediatric ASD studies, natriuretic peptides have been shown to be elevated prior to treatment and decreased afterwards [15,16]. However, such levels were not measured in newborns with ASD nor directly compared to CMR findings. In adults with heart failure, elevated ST2 levels have been shown to predict adverse cardiovascular outcomes independent of natriuretic peptides, while ST2 data on children or adults with CHD is currently limited [1,3,17,18]. Because CHD is the most common organ anomaly, affecting approximately 1:125 newborns, this study set out to explore the usefulness of ST2 in the setting of pediatric CHD by focusing on ASD, as this lesion represents a common and clinically important type of CHD that occurs in approx. 1:1000 live births [19,20,21,22]. Higher mortality, compared to the general population, has been reported in patients with ASD, and timely closure of the defect in childhood positively affects long-term outcomes [23]. Chronic volume loading of right-sided heart structures in ASD patients can lead to exercise intolerance, pulmonary hypertension, and heart failure, but these may initially go unnoticed during early childhood as clinical symptoms may be mild at first, evolve slowly, or may even remain completely absent [24,25]. Though ASD can be diagnosed by echocardiography, slowly evolving cardiac dysfunction, which may negatively impact outcomes, may be challenging to recognize by echocardiography [26,27,28]. Here, CMR offers advantages when assessing left- and right-sided ventricular systolic function [29,30].

Because no previous study has specifically assessed ST2 blood levels in newborns with ASD, we quantified ST2 using a dried blood spot assay, similarly to tests commonly used in newborn screening programs. We hypothesized that dried blood spot levels of ST2 in newborns with ASD, who later presented with symptoms and required defect closure during childhood, would differ from those seen in newborn controls and that ST2 measurements could potentially aid early diagnosis.

We further aimed to evaluate the complementary role of ST2 during CMR assessments in pediatric ASD and measured venous blood ST2 concentrations in children before and after ASD treatment. Blood sampling was timed to CMR scans, allowing for a direct comparison of ST2 blood levels and cardiac imaging findings in cases and matched controls. We hypothesized that higher levels of ST2 in pediatric ASD would be associated with lower levels of cardiac function on CMR and that ST2 levels should decline after ASD closure, in line with the overall normalization of atrial shunt flows and corresponding changes to ventricular loading conditions. This could make ST2 a potential additional biomarker to monitor cardiovascular remodeling following CHD treatment or even help to characterize evolving myocardial dysfunction in children.

## 2. Results

**Figure 1:**
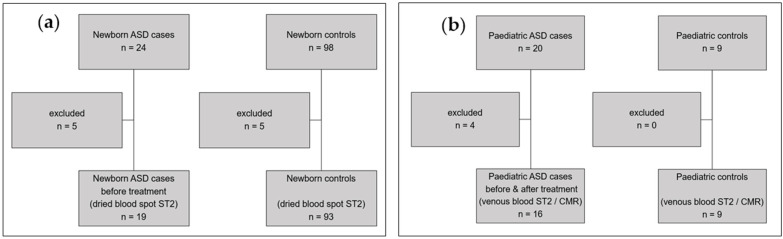
(**a**,**b**): Study participants of newborn ASD cases versus newborn controls and pediatric ASD cases versus pediatric controls. (**a**): Dried blood spot ST2 analysis in 19 newborn ASD cases versus 93 newborn controls. Exclusions due to insufficient dried blood spot samples. (**b**): Venous blood ST2 analysis and CMR imaging in 16 pediatric ASD cases before and after treatment versus 9 pediatric controls. Exclusions due to non-attendance on follow-up.

Figure 1a,b shows study participants. In the newborn group, 122 infants were recruited and 112 (91.8%) included in the study. In the pediatric group, 29 children were recruited and 25 (83.3%) included. Exclusions were due to insufficient dried blood samples in newborn ASD cases (*n* = 5) and newborn controls (*n* = 5), as well as non-attendance of follow-up assessment in pediatric ASD cases (*n* = 4).

### 2.1. Dried Blood Spot ST2 Analysis in Newborns with ASD

**Figure 2:**
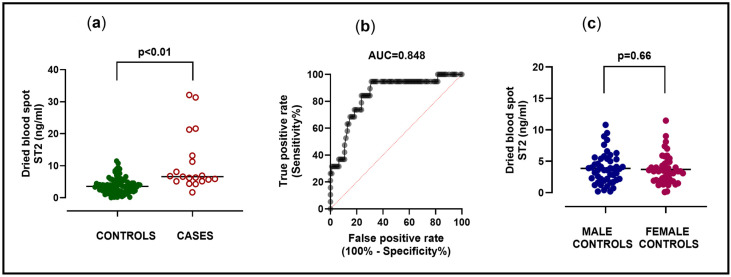
(**a**–**c**): Dried blood spot ST2 analysis in newborn ASD cases versus newborn controls. (**a**): Group comparison of dried blood spot ST2 analysis in 93 newborn controls (median [IQR]: 3.56 [1.97–4.93] ng/mL) versus 19 newborn ASD cases (median [IQR]: 6.60 [5.17–13.18] ng/mL) with significant difference (Mann–Whitney-U test: *p* < 0.01). (**b**): Receiver operating characteristics (ROC) curve of dried blood spot ST2 analysis used to detect ASD amongst 19 newborn cases versus 93 newborn controls showing an area under the curve (AUC) of 0.848, SE 0.048 (95% CI: 0.75–0.94), *p* < 0.01. (**c**): Group comparison of dried blood spot ST2 analysis in 48 newborn males (median [IQR]: 3.70 [2.05–5.38] ng/mL) versus 45 newborn females (median [IQR]: 3.40 [1.95–4.75] ng/mL) without sex difference (Mann–Whitney-U test: *p* = 0.661).

In 19 newborn ASD cases (4 males, 21.1%), dried blood spot ST2 levels were analyzed and compared to 93 newborn controls (48 males, 51.6%). Amongst newborn ASD cases, ST2 levels were higher with a median [IQR] of 6.60 [5.17–13.18] ng/mL compared to 3.56 [1.97–4.93] ng/mL in controls (*p* < 0.01) (Figure 2a). Exclusion of four potential outliers, with the highest ST2 levels amongst cases, did not affect the level of significance (*p* < 0.01). ROC curve analysis to diagnose ASD cases by dried blood spot ST2 levels in this group showed an area under the curve (AUC) of 0.848 (SE: 0.048/95% CI: 0.75–0.94; *p* < 0.01) (Figure 2b). Table 1a gives an overview on how different ST2 cut-off levels would have affected sensitivity, specificity, and likelihood ratios for the dried blood spot test to diagnose ASD in this group of newborns. Newborn characteristics are summarized in Table 1b. Amongst these, timing of dried blood spot sampling was comparable in cases and controls (*p* = 0.711). We observed female predominance amongst newborn ASD cases compared to controls (*p* = 0.021). To check for possible sex-related differences of ST2, dried blood spot ST2 levels in 48 male and 45 female controls were compared, which showed a median [IQR] of 3.70 [2.05–5.38] ng/mL in boys, versus 3.40 [1.95–4.75] ng/mL in girls, without a measurable sex difference (*p* = 0.661) (Figure 2c). No cardiac diseases were documented in newborn controls during follow-up. All 19 newborn ASD cases underwent treatment during childhood, with ASD device closures during cardiac catheterization occurring in 13/19 (68.4%) cases. In 6/19 (31.6%) cases, cardiac surgery was performed, of which 2/19 (10.5%) had associated partial anomalous pulmonary venous return.

### 2.2. Venous Blood ST2 Analysis in Children with ASD

**Figure 3:**
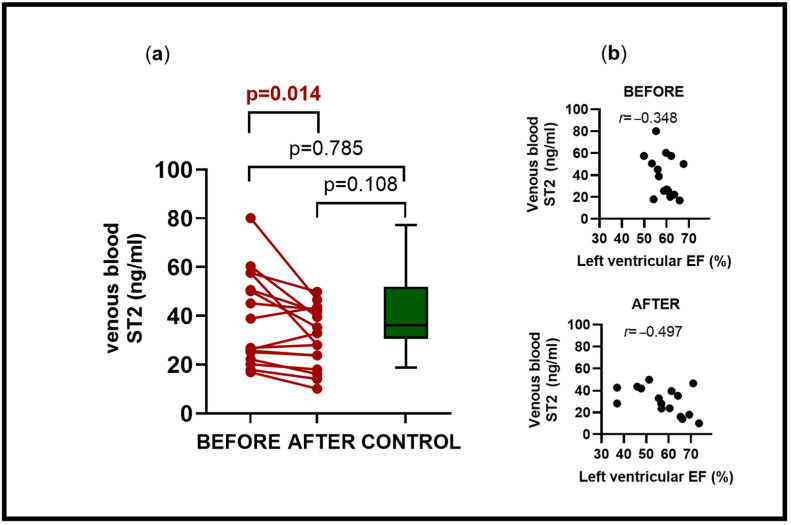
(**a**,**b**)**:** Venous blood ST2 analysis and left ventricular ejection fraction in pediatric ASD cases before and after treatment. (**a**): Venous blood ST2 analysis in 16 pediatric ASD cases before treatment showed a mean ± SD 38.84 ± 18.95 (95% CI: 28.74–48.94) ng/mL versus 30.88 ± 12.48 (95% CI: 24.23–37.53) ng/mL after treatment with significant difference (Student’s paired *t*-test: *p* = 0.014). Comparison of cases before and after treatment versus controls (Student’s unpaired *t*-tests: before vs. controls *p* = 0.785; after vs. controls *p* = 0.108). (**b**): Relationship of venous blood ST2 levels and left ventricular ejection fraction (EF).

Table 2a summarizes venous blood ST2 findings and CMR results in pediatric cases and controls. Sixteen pediatric ASD cases (7 males; 43.7%), in which venous blood ST2 levels were measured together with CMR imaging before and after ASD treatment, were compared to 9 matched controls (4 males, 44.4%). Venous blood concentrations in these pediatric ASD cases decreased from 38.84 ± 18.95 (95% CI: 28.74–48.94) ng/mL before treatment to 30.88 ± 12.48 (95% CI: 24.23–37.53) ng/mL afterwards (*p* = 0.014) (Figure 3a). Higher venous blood ST2 levels in ASD cases correlated with lower left ventricular (LV) systolic function, as measured by ejection fraction (EF), before treatment (r = −0.348) and afterwards on follow-up (r = −0.497) (Figure 3b). There was no difference in sex distribution between pediatric cases and controls (*p* = 0.975). Both groups had comparable age (*p* = 0.430), heart rates (*p* = 0.156), and BSA at study enrolment (*p* = 0.408) (Table 2b). No arrhythmias were documented on ECG, and no additional cardiovascular comorbidities or other health condition were identified in cases and controls. In 13/16 (81.2%) of cases, ASD was closed using devices during cardiac catheterization. Three (18.8%) children required cardiac surgery, of which one (6.3%) had additional partial anomalous pulmonary venous return. Repeat assessment in cases was at 7.73 ± 1.73 months following treatment. Atrial shunt ratios (Qp:Qs) normalized from a median [IQR] of 1.77 [1.55–2.65] to 1.02 [0.94–1.10] (*p* < 0.01), indicating successful removal of shunt flows. For the left ventricle, indexed end-diastolic and end-systolic volumes as well as indexed LV stroke volumes in cases were smaller before treatment compared to controls (all: *p* < 0.01) and increased to normal levels afterwards. Cardiac index (CI) remained stable and within normal ranges in cases before and after ASD treatment (*p* = 0.170). Systolic LV function assessed by ejection fraction (EF) remained stable in the ASD group before and after treatment (*p* = 0.540) with only four patients showing a decline in EF after treatment. Overall, EF in the ASD group was comparable to controls before (*p* = 0.547) and after treatment (*p* = 0.919). For the right ventricle, indexed end-diastolic and end-systolic volumes, as well as stroke volumes, were larger before ASD closure in cases compared to controls (all: *p* < 0.01). Right ventricular (RV) volumes reduced in cases when comparing results before and after ASD treatment (all: *p* < 0.01), with end-systolic RV volumes remaining larger on follow-up in cases compared to controls (*p* = 0.036). Systolic RV function assessment showed stable EF findings before versus after ASD treatment in cases (*p* = 0.086). RV EF in cases was comparable to controls before (*p* = 0.986) and after treatment (*p* = 0.102).

## 3. Discussion

This explorative case–control study of ST2 levels in newborns with ASD showed that ST2 dried blood spot levels were higher in cases compared to controls with good test differentiation, as shown by an AUC of 0.848 on ROC analysis. As circulatory changes in ASD are expected to evolve gradually after birth, newborns with ASD would typically be asymptomatic, making an early clinical diagnosis difficult. Here, ST2 assessment may offer additional value by potentially identifying young ASD cases using dried blood spot analysis, thereby offering additional screening methods for the early diagnosis of ASD. Physiological changes lead to gradually increased pulmonary blood flow and volume loading of right-sided heart structures in young patients with ASD, leaving the left ventricle relatively underfilled [31,32]. These pathophysiological effects on circulation make ASD a suitable model to study ST2 because levels may reflect myocardial as well as pulmonary circulatory changes [6,7]. The elevated ST2 levels in newborn ASD in this study may reflect circulatory adaptations that lead to the release of ST2 due to mechanical stress on cardiomyocytes or due to release from pulmonary circulation [33,34]. Future studies in newborns should evaluate the usefulness of ST2 as a potential screening test for this common type of CHD, as well as its role in other types of heart disease affecting ventricular loading conditions soon after birth.

Normal ranges for ST2 using serum or plasma blood samples in adults have been reported [35,36,37]. Sex and age differences of ST2 levels have also been described in adults with coronary heart disease, myocarditis, and cardiomyopathies [38,39]. In this study, we observed more girls than boys in our newborn ASD group, which is in line with published reports showing female ASD predominance [21,40,41]. Because of the observed higher proportion of female newborns with ASD, we confirmed comparable dried blood spot ST2 levels for both sexes in our newborn controls. This is in line with published pediatric data showing comparable ST2 levels in boys and girls [42]. Because published normative reference data (using the applied dried blood spot and venous ST2 assays) from large newborn cohorts are currently lacking, this study could serve as a baseline to establish more ST2 reference data.

In other clinical settings, higher ST2 blood levels in children have been associated with unplanned hospital admissions following open-heart surgery [43]. We recorded no unplanned hospital admissions after pediatric ASD closures, albeit most patients in this study had ASD device closures during cardiac catheterization rather than open-heart surgery. Pre-existing comorbidities that have been associated with elevated ST2 levels were not documented in our ASD group, such as congestive heart failure, chronic systemic inflammatory processes, asthma, other chronic lung disease, obesity, hypertension, diabetes mellitus, or certain cancer types [44,45,46,47,48,49,50]. Neither documented arrhythmias nor pulmonary hypertension were apparent, which may otherwise impact outcomes in ASD patients [51,52,53]. All pediatric cases in this study required ASD intervention during childhood based on standard guidelines [54]. With these clinical management decisions made prior to study enrolment, successful ASD closure could be confirmed by normalization of atrial shunt ratios on CMR during follow-up. We saw a decline of EF in a minority of four ASD cases on follow-up after changes to LV loading conditions and observed larger RV end-systolic volumes in cases after treatment compared to controls, which together may reflect ongoing cardiac remodeling in at least some patients that would have warranted longer-term evaluation than this study was designed for. We documented increases in LV volumes with CI remaining within normal range after treatment, which suggests cardiac benefits, as reported in other ASD studies [55,56]. Because higher venous blood ST2 levels in pediatric ASD cases were associated with lower EF of the left ventricle before and after treatment, ST2 measurements may complement LV functional assessment if these findings could be confirmed in additional studies. This could potentially assist in the evaluation of children at risk of evolving LV systolic dysfunction. These findings underline the potential role of ST2 assessments in children that is also emerging from other reports studying various pediatric heart conditions [8,57,58]. From a physiological point of view, the previously underfilled left ventricle usually adapts after ASD treatment by normalizing in size [56]. Associated normalization of biomechanical stress on cardiomyocytes paired with normalization of pulmonary blood flow should, in this context, lead to decreased ST2 levels, as observed in our studied children following ASD closure. However, in some patients who may have a less compliant left ventricle following the chronically underfilled preoperative state, LV function may be challenged by the increased volumes after ASD closure and ST2 levels may be higher due to persistent LV biomechanical stress. Therefore, future studies should address the questions of how elevated ST2 blood levels may be causally related to pathophysiological changes in children with various types of heart disease, and how ST2 measurements may aid treatment assessments and evaluation of long-term outcomes.

Although no clinical signs of inflammation were documented amongst children in this study and only small blood sample quantities were available with a predefined focus on ST2 analysis, future studies should consider additional analyses of inflammatory biomarkers in parallel to ST2 to gain more insights into possible inter-relationships between cardiac pathology and inflammation, since previous studies concerning inflammatory conditions have described increased ST2 levels in children and adults [57,59,60].

As ASD referral patterns or institutional treatment practices may vary in other clinical settings and study participants were recruited by non-random methods, this should be considered when interpreting findings. Without a prior power calculation, the study may have been prone to lower statistical power and higher risk of type II error, which might have led to incorrectly concluding that there was no difference in certain measures due to, e.g., a small sample size or sample variability. Larger prospective multi-center studies should allow for an evaluation of ST2 alongside other cardiovascular biomarkers, such as natriuretic peptides, together with CMR and other comprehensive cardiac imaging modalities, to determine whether ST2 has additional clinical value in the management of ASD and other pediatric and adult CHD patients with additional risk factors, such as pulmonary hypertension or heart failure.

### Limitations

This study was designed as an explorative study to evaluate the usefulness of ST2 in a novel context of pediatric CHD. It included a relatively small sample size of newborns and children, with ASD assessed at a single center. In enrolled newborns, timing of dried blood spot sampling was comparable in ASD cases and controls, and we could show that sex differences in groups were unlikely to influence ST2 levels. However, the retrospective character of this study partly limited our ability to account for other perinatal factors due to the lack of available clinical data that could have influenced ST2 levels, e.g., in the case of maternal gestational diabetes [61]. To reduce the risk for potential confounders affecting results in pediatric ASD cases and controls, we matched study participants according to sex, age, heart rates, BSA and used established CMR methods to compare ST2 blood levels against cardiac imaging findings during prospective follow-up of children after ASD treatment.

Although blood levels of natriuretic peptides have been evaluated in children and adults with structural and functional heart disease and may give insights into the pathophysiological processes affecting the circulatory system, previous data on children with ASD suggest limited usefulness in this setting [33,62,63,64,65,66,67,68]. Because we had access to only small amounts of dried blood spot and venous blood samples, we focused our biomarker analysis on ST2 in this study, with the applied assay previously showing a good correlation between dried blood spot and venous blood EDTA samples [69]. Levels of ST2 in frozen blood samples have been reported to be stable, and serum and plasma levels are generally comparable [70,71]. With no published evidence to suggest that red blood cells interfere with ST2 measurements, dried blood spot ST2 levels should reflect those seen in serum or plasma samples. As there is currently no published data with a suitable correction factor derived from larger sample matrixes for the dried blood spot and venous blood ST2 tests, assay results were not directly comparable, underscoring the need for standardized ST2 measurements [72]. Due to these limitations, caution should be used when interpreting study findings before generalization can be applied.

## 4. Materials and Methods

The study was conducted in Sweden between August 2020 and January 2025. Following approval by the Swedish National Ethical Review Authority (2019-05490), we followed the principles of the Declaration of Helsinki [73]. The study was registered on the Clinical Trials website (NCT04667455) and reported according to STROBE guidelines [74]. Written, informed consent was obtained from guardians, with the child’s assent sought where possible. Using an explorative case–control study design to measure ST2 in newborns and older children with ASD, we aimed for a minimum case to control ratio of 1:2 with no formal power calculations performed, as there were no previously pediatric ASD data available. Eligible CHD diagnoses in cases were ASD, including those with partial anomalous pulmonary venous return. This was based on diagnosis codes Q20 through to Q28 of the International Statistical Classification of Disease and Related Health Problems; Tenth Revision. Cases with ASD and partial anomalous pulmonary venous return were analyzed together with other ASD cases as this additional diagnosis contributed parts of the overall pathophysiological volume-loading effect to right-sided heart structures in this setting. We verified diagnoses and treatments against electronic medical notes, with additional review of cardiac imaging findings in those children who underwent CMR scans as part of this study. To reflect usual clinical practice of the newborn screening program in the studied setting, dried blood samples had to be taken within the first week of life for study eligibility. All newborns had to be born at term without documented neonatal treatment requirements and without recorded clinical symptoms of heart disease in the newborn period. Recruitment for analysis of dried blood spot samples in newborn ASD cases and controls followed non-random methods. Newborn cases were enrolled after ASD diagnosis was established during childhood following pediatric cardiology assessments. Newborn controls were enrolled during neonatal check-ups or during later pediatric cardiology out-patient clinic visits that documented normal cardiac findings. Newborn controls were followed up for one year using electronic records to ensure no clinical signs of heart disease developed. We recorded sex and the day of life on which the newborn dried blood spot samples were taken.

To additionally assess ST2 biomarker levels and CMR imaging findings in pediatric ASD, children admitted for ASD treatment at a single tertiary pediatric cardiac center were enrolled in order of their clinically scheduled appointments. These pediatric ASD patients were identified through existing hospital planning records and recruited on initial admission for ASD treatment with baseline assessments performed on this same visit prior to ASD closure. These children underwent CMR, with venous blood samples taken at the time of CMR scans. Repeat assessments with CMR and venous blood sampling were performed six to twelve months after ASD treatment in these children. Pediatric controls were recruited following local advertisement and matched to pediatric cases with regard to sex, age, heart rates, and BSA.

At the time of assessments, electronic records were checked for additional health conditions as well as other cardiovascular comorbidities, such as hypertension, arrhythmias on electrocardiograms (ECG), pulmonary hypertension, or valvar heart disease.

Measurements of ST2 levels were performed using a previously developed assay [69]. The coefficient of variation for the ST2 assay with dried blood spot samples was determined to be 5.2% with a limit of detection 0.19 ng/mL and limit of quantification 0.63 ng/mL. Assay calibrator values were assigned against concentration of purified recombinant antigen. The main limiting factor between the venous blood samples and dried blood spot samples was the available small sample volume, which was approximately 3 µL of whole blood from dried blood spot punches of which approximately 1.5 µL was serum. With laboratory staff blinded to clinical data, batched analyses of blood samples were performed after +4 °C storage for dried blood spot samples and after −80 °C storage of venous ethylenediaminetetraacetic acid (EDTA) blood samples.

CMR was performed using a 1.5 Tesla scanner (Aera, Siemens Healthineers, Erlangen, Germany) with acquisition of balanced steady-state free precession (bSSFP) short-axis cine images covering the whole heart with retrospective ECG-gating. Two-dimensional free-breathing through-plane phase-contrast flow was measured in the ascending aorta and main pulmonary artery to quantify stroke volumes and shunt ratios (Qp:Qs). After ventricular delineations on short-axis bSSFP stacks, cardiac volumes were assessed [75]. One experienced examiner (PS) checked CMR data prior to analyses, ensuring adequate image quality was obtained. We accounted for variable body sizes in children by indexing CMR measurements to body mass index (BSA) using Mosteller’s method and analyzed data with Segment software, version 4.0 R11026 (Medviso AB, Lund, Sweden).

We used statistical software to analyze data (GraphPad Prism version 10.2.1, Boston, MA, USA). After checking data distribution, using visual data inspection and Shapiro–Wilk tests, results were expressed in whole numbers (percentage), using mean ± SD for parametrically or median [IQR] for non-parametrically distributed data. As an explorative study, no adjustments for potential multiple comparisons of continuous variables were made. Sub-group analysis was performed for newborn controls to assess sex distribution of ST2. To compare newborn ASD cases to newborn controls and pediatric ASD cases before and after treatment to pediatric controls, we applied unpaired Student’s *t*-tests for parametrically or Mann–Whitney-U tests for non-parametrically distributed data. For group comparison of pediatric ASD cases on initial versus follow-up visits, paired Student’s *t*-tests or Wilcoxon tests were used for parametrically or, respectively, for non-parametrically distributed data. Receiver operating characteristics (ROC) curve analysis was used to evaluate dried blood spot blood test performance in newborns to detect ASD cases. In the pediatric ASD cohort, Pearson correlation analysis was used to assess the relationship between ST2 blood concentrations and left ventricular (LV) ejection fraction (EF), derived from CMR. We accepted two-sided *p*-values < 0.05 as statistically significant.

## 5. Conclusions

Dried blood spot concentrations of ST2 were higher in newborns with ASD compared to controls, which may aid early diagnosis of this common, but often asymptomatic type of CHD in the young. After pediatric ASD treatment, venous blood levels of ST2 decreased, whilst higher ST2 levels were associated with lower LV systolic heart function as assessed by EF. Findings underscore the potential of ST2 in evaluating ASD treatment during childhood and its possible role in unmasking evolving LV dysfunction. Results warrant further studies to evaluate the usefulness of ST2 for early diagnosis of this and other types of CHD, as well as ST2’s role during clinical follow-up in children, to potentially guide treatment decisions. Even though ST2 has been implicated in a wide spectrum of pathophysiological states and diseases, its role as a diagnostic or prognostic cardiovascular biomarker in children appears to be still emerging. Future research may also assess ST2 as a therapeutic target to modulate inflammatory or fibrotic processes within circulation to positively influence clinical outcomes in patients with heart disease.

## Figures and Tables

**Table 1:** (**a**) Overview of how different ST2 cut-off levels (ng/mL) using dried blood spot analysis in newborns would affect detection of ASD in this study. (**b**) Characteristics of newborns with ASD versus newborn controls assessed by dried blood spot ST2 analysis.

(**a**)					
**ST2 Level (ng/mL)**	**Sensitivity**	**95% CI**	**Specificity**	**95% CI**	**Likelihood Ratio**
>4.28	94.74	75.36% to 99.73%	69.89	59.93% to 78.27%	3.15
>4.61	84.21	62.43% to 94.48%	72.04	62.19% to 80.15%	3.01
>4.93	84.21	62.43% to 94.48%	75.27	65.62% to 82.92%	3.41
>5.24	73.68	51.21% to 88.19%	76.34	66.77% to 83.83%	3.12
>5.64	73.68	51.21% to 88.19%	81.72	72.66% to 88.26%	4.03
>5.89	68.42	46.01% to 84.64%	84.95	76.30% to 90.82%	4.55
>6.45	52.63	31.71% to 72.67%	88.17	80.05% to 93.27%	4.45
>7.21	36.84	19.15% to 58.96%	90.32	82.62% to 94.82%	3.81
>8.70	31.58	15.36% to 53.99%	94.62	88.03% to 97.68%	5.87
(**b**)					
**Newborns** **(*n* = 112)**					**Statistical Difference**
			Newborn Controls (*n* = 93)	Newborn Cases (ASD) (*n* = 19)	Newborn Controls versus Newborn Cases
Sex					
female *n* (%)			45 (48.4)	15 (78.9)	*p* = 0.021 ^#^
male *n* (%)			48 (51.6)	4 (21.1)	
Day of life for dried blood spot sampling					
mean ± SD (95% CI)			2.72 ± 0.71 (2.57–2.87)	2.79 ± 0.86 (2.38–3.20)	*p* = 0.711 ^#^
Treatment by					
device closure *n* (%)			N/A	13 (68.4%) 6 (31.6%)	N/A
open-heart surgery *n* (%)					

Results are stated in whole numbers (%) for sex and treatment by device or open-heart surgery. Results are expressed as mean ± SD for day of life for dried blood spot sampling. ^#^ Student’s unpaired *t*-tests were used to compare sex and days of life for dried blood spot sampling in cases versus controls. Abbreviation in table: N/A: not applicable.

**Table 2:** (**a**) Venous blood ST2 levels and CMR imaging in pediatric ASD cases before and after treatment versus pediatric controls. (**b**) Characteristics of pediatric ASD cases versus pediatric controls assessed by venous blood ST2 analysis and CMR imaging.

(a)						
	ASD Before Treatment (*n* = 16)	ASD After Treatment (*n* = 16)	ASD Before Treatment Versus ASD After Treatment	Controls (*n* = 9)	ASD Before Treatment Versus Controls	ASD After Treatment Versus Controls
**Venous blood ST2 level** mean ± SD (95% CI)						
Venous blood ST2 level, ng/mL	38.84 ± 18.95 (28.74–48.94)	30.88 ± 12.48 (24.23–37.53)	*p* = 0.014 *	40.97 ± 17.57 (27.46–54.47)	*p* = 0.785 *	*p* = 0.108 *
**CMR of the left ventricle** mean ± SD (95% CI)						
LV EDVi mL/m^2^	65.5 ± 11.3 (59.5–71.6)	79.6 ± 15.5 (71.3–87.8)	*p* < 0.01 *	83 ± 8 (77–88)	*p* < 0.01 *	*p* = 0.705 *
LV ESVi mL/m^2^	26.9 ± 5.9 (23.7–30.0)	34.4 ± 13.0 (27.5–41.3)	*p* < 0.01 *	34 ± 5 (31–38)	*p* < 0.01 *	*p* = 0.987 *
LV SVi mL/m^2^	38.7 ± 6.9 (35.0–42.3)	45.2 ± 9.6 (40.0–50.3)	*p* = 0.020 *	48 ± 7 (44–53)	*p* < 0.01 *	*p* = 0.555 *
LV EF %	59.1 ± 4.7 (56.6–61.6)	57.4 ± 11.2 (51.5–63.4)	*p* = 0.540 *	56 ± 6 (52–60)	*p* = 0.547 *	*p* = 0.919 *
CI L/minute/m^2^	3.26 ± 0.65 (2.91–3.61)	3.59 ± 0.88 (3.12–4.06)	*p* = 0.170 *	3.68 ± 0.60 (3.22–4.14)	*p* = 0.127 *	*p* = 0.801 *
**CMR of the right ventricle** mean ± SD (95% CI)						
RV EDVi ml/m^2^	141.4 ± 40.4 (119.9–162.9)	95.7 ± 22.2 (83.9–107.6)	*p* < 0.01 *	84.9 ± 8.5 (78.3–91.4)	*p* < 0.01 *	*p* = 0.175 *
RV ESVi mL/m^2^	61.0 ± 22.6 (49.0–73.1)	49.4 ± 17.5 (40.1–58.7)	*p* < 0.01 *	36.0 ± 5.2 (32.0–40.0)	*p* < 0.01 *	*p* = 0.036 *
RV SVi mL/m^2^	80.6 ± 20.8 (69.5–91.7)	46.2 ± 12.5 (39.5–52.9)	*p* < 0.01 *	48.8 ± 7.0 (43.4–54.2)	*p* < 0.01 *	*p* = 0.569 *
RV EF %	57.6 ± 6.1 (54.4–60.8)	50.4 ± 12.5 (44.0–56.7)	*p* = 0.086 *	57.6 ± 5.3 (53.5–61.7)	*p* = 0.986 *	*p* = 0.102 *
**CMR-derived atrial shunt ratio** median [IQR]						
Qp:Qs	1.77 [1.55–2.65]	1.02 [0.94–1.10]	*p* < 0.01 **	1.01 [1.00–1.06]	*p* < 0.01 **	*p* = 0.890 **
**(b)**						
**Children** **(*n* = 25)**						**Statistical difference**
		**Pediatric Controls** **(*n* = 9)**		**Pediatric Cases** **(ASD)** **(*n* = 16)**		**Pediatric Controls** **Versus** **Pediatric Cases**
Sex						
female: *n* (%) male: *n* (%)		5 (55.6) 4 (44.4)		9 (56.3) 7 (43.7)		*p* = 0.975 ^##^
Age (years)						
mean ± SD (95% CI)		8.3 ± 1.7 (7.0–9.7)		9.6 ± 4.4 (7.2–11.9)		*p* = 0.430 ^##^
Body surface area (m^2^)						
mean ± SD (95% CI)		1.22 ± 0.48 (0.96–1.47)		1.07 ± 0.16 (0.95–1.20)		*p* = 0.408 ^##^
Heart rate (bpm)						
mean ± SD (95% CI)		78 ± 8 (72–84)		86 ± 15 (78–93)		*p* = 0.156 ^##^
Follow-up (months)						
mean ± SD (95% CI)		N/A		7.73 ± 1.73 (6.82–8.65)		N/A
Treatment by						
device closure *n* (%) open-heart surgery *n* (%)		N/A		13 (81.2) 3 (18.8)		N/A

* Results are expressed as mean ± SD (95% CI). Student’s paired *t*-tests were used to compare ASD cases before versus after treatment. Student’s unpaired *t*-tests were used to compare ASD cases before and after treatment versus controls. ** Results are expressed as median [IQR]. Mann–Whitney-U tests were used to compare ASD cases before versus after treatment. Mann–Whitney-U tests were used to compare ASD cases before and after treatment versus controls. Abbreviations in table: ASD: atrial septal defect; BSA: body surface area; CI: cardiac index; EDVi: end-diastolic volume indexed for BSA; EF: ejection fraction; ESVi: end-systolic volume indexed for BSA; LV: left ventricular; N/A: not applicable; Qp: pulmonary blood flow; Qs: aortic blood flow; RV: right ventricular; SVi: stroke volume indexed for BSA. Results are expressed as whole numbers (%) for sex and treatment by device closure or open-heart surgery. Results are expressed as mean ± SD for age, BSA, heart rate and follow-up. ^##^ Student’s unpaired *t*-tests were used to compare cases versus controls.

## Data Availability

Data request can be made to the authors from non-profit/academic institutions and will be released after approval from the authors as deidentified data for research purposes if in line with the legal and ethical framework of this study.

## Abbreviations

ASD	atrial septal defect
AUC	area under the curve
BSA	body surface area
bSSFP	balanced steady-state free precession
CHD	congenital heart disease
CMR	cardiac magnetic resonance
ECG	electrocardiogram
EDTA	ethylenediaminetetraacetic acid
EF	ejection fraction
IQR	interquartile range
LV	left ventricular
N/A	not applicable
NT-proBNP	amino-terminal prohormone of brain natriuretic peptide
Qp	pulmonary blood flow
Qs	systemic blood flow
ROC	receiver operating characteristics
RV	right ventricular
SD	standard deviation
SE	standard error
ST2	soluble growth stimulation protein form of interleukin-1 receptor-like 1
95% CI	95% confidence interval
